# Address Delivered before the Ill. State Med. Society

**Published:** 1851-09

**Authors:** W. B. Herrick

**Affiliations:** President of the Society, Professor of Anatomy and Physiology in Rush Medical College, &c., &c.


ARTICLE VIII.

*Address, on the Remedial Properties of Alimentary Substances, and the Changes produced by Oxygen in Health and Disease.* Delivered before the ILLINOIS STATE MEDICAL SOCIETY. By W. B. HERRICK, M. D., President of the Society, Professor of Anatomy and Physiology in Rush Medical College, &c., &c.

[Taken from the transactions of said Socieety.]

GENTLEMEN:—

This, the first annual meeting after that for the organization of the Illinois State Medical Society, is an occasion of great interest and of much importance.

It is interesting, from the fact that the nuber here present furnishes abundant evidence of the continued growth and prosperity of an organized body, the noble object of which is to combine and render efficient in action the talent and enterprise of our medical men, and to cherish among them feelings of brotherly love and friendship. Importat, because our present meeting furnishing, as it does, the only opportunity which has ever been offered for the united action of any considerable number of our physicians, must, to a certain extent, give character, both at home and abroad, to the medical profession of our State.

The principal object of an organization like ours should be, doubtless, to furnish a medium through which medical men can reciprocally make known to each other the results of their individual observation and experience, in order that the knowledge acquired by each in their respective localities, may here become the common property of all. With the view of sustaining my part in the performance of this common duty, and in compliance with a requisition in the constitution of this Society, requiring me to deliver an address at the expiration of my term of office, I will now endeavor to perform this, a pleasant task, generously assigned me by your partiality and kindness.

On an occasion not long since, upon which I had the honor of addressing the public in behalf of our profession, it was stated as my opinion, that "the time is not far distant when the truly scientific physician will use as remedies such substances only as help to constitute in health the solids and fluids of the body."

We had arrived at this conclusion after mature reflection upon the present state of our science, and after having observed a tendency on the part of both writers and practitioners of the present day, to regard the manifestations of disease in the human body as evidences of want of harmony in the performance of functions consequent upon excess or deficiency in some of its parts or elements which may be, and are often, more promptly restored by the addition or abstraction of one or more of its normal and proper constituents, rather than by introducing into the system, powerful and even poisonous foreign substances.

Believing, as we do, that this is the truly scientific and rational view to take of disease, and remedial agents, and that the spirit thus manifesting itself is one of improvement, indicating the approach of a new era in the study and practice of medicine, we propose on the present occasion to lay before the Society our views of the nature of certain chemico-vital changes, and of the modus operandi of certain medicines with the view of showing, what we firmly believe, that a majority of diseases may be cured more certainly and promptly by the use of properly chosen alimentary substances than by the administration of powerful agents under the false name of remedies; and that the animal oils—lemon juice, common salt, soda, &c.—are valuable and effective medicines, capable of restoring health in some of the worst forms of disease; and that in view of certain changes in the body, especially such as are effected by the oxygen of the air, the physiological reasons for their action, and pathological indications for their use, are plain and rational.

The views expressed by most physiological writers concerning the changes resulting from the action of oxygen in the animal body are, to say the least, vague and unsatisfactory.

According to the genarally received opinion, it is taken into the system for the purpose of being combined with the nutritious materials derived from food, to form the more highly organized and perfectly animalized constituents of the blood and tissues.

In opposition to this view, we contend that digestion and respiration are antagonistic in their functions; that the most highly elaborated nutritious substances may be derived directly from food, in a form suitable to enter at once into the constitution of the most perfect organs; and that all the constituents, both of the blood and tissues, by uniting with oxygen, are passing continually into less perfectly organized forms, and hence that oxygenation is a destructive, rather than a vitalizing process. To use the words of B. Jones, " a change of one substance into a higher compound never occurs, but everywhere in the stomach and system lower compounds are each moment forming, and this chiefly for the purpose of solution." All the phenomena, both of healthy and diseased action, are more fully and satisfactorily explained by thus considering oxygen the agent by means of which organic matter is reduced and ultimately separated from the body, as it is well known it is, in the form of carbonic acid, urea uric, acid, &c., all highly oxygenized compounds.

According to the views of Prof. Liebig, certain constituents of the food, known as the non-nitrogenized or carbonaceous, are taken into the system for the purpose, principally, of gererating animal heat, by their union with oxygen; whilst others, the nitrogenized or albuminous, furnish mainly nutriment for the organs and tissues.

Admitting the correctness of these views, it is evident that in case of deficiency of carbonaceous substances with an excess of oxygen in the system, the changes which would be most likely to occur would be of such a nature as to give rise to a class of diseases characterized by excessive oxydation, whilst an opposite condition as regards the relative proportions of oxygen and oxegisical materials might produce other affections no less numerous, in which defective oxidation would be the leading characteristic.

Among those belonging to the first class are, as we believe, all highly inflammatory affections—such as Pneumonia, Pleurisy, Croup, &c., together with Rheumatism, Tubercular disease, and most affections of the skin, all of which are most common and severe in high northern latitudes, where a dense and dry atmosphere favors excessive oxidation; and of an opposite character to those which prevail in the south, where the rare and humid air, deficient in oxygen, gives rise to Yellow, Remittent and Intermittent fevers, Jaundice, malignant Erysipelas, &c., in which there is doubtless an accumulation of unoxidized effete matter in the blood and tissues.

The physiological and pathological facts which might be adduced in support of the above conclusions, are too numerous to admit of being presented on the present occasion. The most we can hope to accomplish at this time, is to give a few reasons and present some facts, such as may serve to direct the attention of medical men to the subject, and show practitioners the propriety of depending more upon remedial agents, which modify in health these changes, and which are therefore not destructive, but congenial to life.

All inflammatory affections are, according to our views, attended by excessive oxydation. This is evident from the increased amount of carbonic acid exhaled, and the constant accumulation in the blood, during their progress, of highly oxygenized compounds, such as urea, uric acid, fibrine, &c. The most remarkable characteristic of this class of diseases, is the constant and rapid increase, during their progress, of the fibrine of the blood. This substance, according to most physiologists, is the more highly organized constituent of this nutritious fluid, formed from its less perfectly animalised ingredient albumen; whilst others, on the other hand, contend that the latter substance as compared with the former, is the most important, and that fibrine of the two, is the most nearly allied to effete matter. The latter opinion is doubtless the correct one, as is evident for several reasons, among which may be mentioned the facts, that fibrine is not an ingredient of the highly nutritious compound provided by nature in the albuminous egg; neither does it enter, in any considerable quantity, as compared with albumen, into the constitution of healthy blood—whilst it accumulates rapidly during the progress of diseases in which there is defective action in the assimilative and excretive functions.

In support of this view we have also the analysis of Muuler, who found more fibrine in the oxygenized arterial than in the carbonized venous blood. The experiments of Marchal, showing an increase of this element in blood drawn from a vein under the influence of heat, such as would favor oxidation; those of Becquerel, by which it appears that albumen diminishes rapidly during the progress of diseases, such as Pneumonia, Pleurisy, &c., in which fibrine increases; and the fact stated by Hassall, in his Microscopic Anatomy, of the human body, where it is asserted that "the true cause of the fatality which has so often attended the operntion of transfusion, depends upon the difference which exists in the qualities of the fibrine in the blood of two different animals, or even of two distinct individuals. This is shown by the fact that the transfusion of blood deprived of its fibrine, is never followed by the serious results to which reference has been made."

Hence we conclude that fibrine is like most other morbid constituents of inflammatory blood, a deleterous compound resulting from excessive oxidation consequent upon causes favoring chemico-vital changes, and that one of the most important indications in the treatment of all inflammatory affections, is to prevent oxidation and the accumulation in the blood of oxygenized substances.

Taking this view of the subject, we can explain more easily and phelosophically, the modus operandi of the remedial agents, which have been found by experience, to be most efficient in this class of diseases.

The amount of oxygen taken into the system, is always in proportion to the number of globules in the blood, as has been shown by numerous experiments; hence it is that venesection, by diminishing the number of these globules and consequently the amount of oxygen in the circulating fluids, diminishes also its power of acting upon the blood and tissues, and thus becomes in the hands of an eastern physician treating the pure Pneumonia, such as occurs in the dense atmosphere and plethoric patients of New England, one of the most prompt and efficient remedies. Whilst here in the West, where this disease is more or less complicated and mixed with those of an opposite class, the miasmatic, the practitioner, who by frequent bleedings, should abstract globules from the impoverished and carbonized blood of his pneumonic patient, would be treating a name, rather than the disease. In all inflammatory affection the rule should be, doubtless, to avoid blood-letting wherever there is a tendency to miasmatic or other diseases, in which there is defective oxidation, and to use it boldly, in cases of an opposite character, in which the blood rich in globules contains no excess of carbon.

The action of Opium, another very efficient remedy in inflammatory affections, can also be made to harmonize with our views; for it must be admitted by all, that in proportion as respiration and the circulation are made sluggish by its use, the changes resulting from oxydation would be less rapid and extensive, and experience shows that its action in subduing this class of diseases is more or less marked, in proportion to its effect upon these functions.

In all cases of local inflammation in which there is impoverished and carbonized blood, as in Billious Pneumonia, Heapatitis, Sub-Acute Gastro Enteritis, and similar diseases of the West and South, opium is by far a better remedy than blood-letting; because, by acting in the manner above stated, it lessons the excessive local oyxdation of albumenous compounds without depriving the blood of its globular element required in such cases during convalescence for the oxydation and elemination of carbonaceous materials. Hence the difference of opinion among physicians as to the comparative value of these two remedies.

The affections in which treatment with the view to prevent oxydation is plainly indicated, and most efficient, are those of the skin, resulting either from injury or from other causes. This is evident from the well known fact, that remedial agents which most effectually exclude the atmosphere from abraded, diseased or injured surfaces, are the best local remedies in such cases. Hence it is that such applications as Collodion and Gutta-percha, prevent the pitting in small-pox, stop the spread of mild erythema, favor the union of wounds, and subdue measurably the pain and inflammation of blisters and burns; and hence it is that inunction with fats and oils has been found, both in this country and in Eurape, one of the best means for allaying the heat and irritation in Scarlitena and Rubeola.

Excessive oxydation of the important constituents of the body, according to our views, may result either from an excess of oxygen, or from a deficiency of hydro-carbonaceous substances, requiring in the one instance the exclusion of oxygen, and in the other the introduction into the system of oxydisable materials. All purely inflammatory affections belong doubtless to the first class, and are therefore threated properly when we protect highly inflamed surfaces from the atmosphere, or abstract blood, and thus lessen the amount of oxygen taken into the system by diminishing the number of its carriers, the globules; whilst those of less active nature, such as Phthisis, Tabes Mesenterica, and the like characterized by defective nutrition, require a good nutricious, albuminous diet to serve as nutriment for the broken down organs and tissues, together with an abundant supply of respiratory food, such as will by its affinity for oxygen, prevent most effectually the further excessive oxydation of the more important constituents of the blood and tissues.

In support of these views, of the effect of hydro-carbonaceous compounds in preventing the destructive oxydation of the albuminous constituents of the body, we have the experiments of Dr. Brecker, showing that the use of sugar, alcohol, wine, &c., diminishes the amount of lithates, phosphates, and other animal excretive matters in the urine; also the fact stated by Dr. Chambers, that the use of food constituted principally of fats and oil, reduces the amount of nitrogenized constituents in all the excretions.

From the above facts, we learn why it is that compounds such as Cod Liver Oil, constituted principally of carbon and hydrogen, are of all others the most useful remedies in diseases in which there is an impoverished condition of the blood, resulting either from excessive oxydation, morbid action, or defective nutrition.

It was our intention to give in this connection, a history of a few of the most important cases treated by myself and others, with the view to prevent oxydation in the manner above stated, by the use of Cod Liver

Oil. But we find that such details cannot be entered into in the limited time allotted to us on the present occasion, and that we can only state what we know from personal observation and experience, that for all chronic affections characterized by defective nutrition, such as Tuburcular Disease of the Lungs, and Mesenteric and other Glands, Chronic Bronchitis, Tumefaction and Ulceration of the Throat or Tonsils, Scrofulous Opthalmia, &c., the Cod Liver Oil is by far the best and most efficient remedy known, especially when used in connection with exercise in the open air, and a good nutritious diet, to the exclusion of cathartics, calomel, and other debilitating agents, worse than useless in this class of diseases.

The organic acids constitute another class of simple, yet in many cases active remedies, nearly allied to the animal oils, both in their application and mode of action. This is what might be anticipated in view of the following facts, stated by Prof. Liebig, in his recent work on the Chemistry of Food: "From analysis," says he, "it appears that the non-nitrogenized acid, occurring in the animal organism, is identical with the acid formed in milk when it becomes sour, and into which sugar of milk, starch, grape sugar and cane sugar, are converted by contact with animal substances in the state of decomposition." Concerning the use of this acid he says: "The urine of healthy men, which has an acid reaction, contains no lactic acid, and no substance from which lactic acid can be formed during putrefaction of urine." * * "From this it plainly appears, that the lactic acid, in the organism, is employed to support the respiratory process, and the function performed by sugar, starch, and in general all these substances which in contact with animal matter, are convertable into lactic acid, ceases to be an hypothesis. These substanees are converted, in the blood, into lactates, which are destroyed as fast as they are produced, and which only accumulate when the supply of oxygen is less, or where some other attraction is opposed to the agency of that element."

In view of the above facts, it is evident that the use of the organic acids, especially the lactic, is indicated in diseases such as Rheumatism, Gout, &c., in which there is an excess of lithic, oxalic, and other excrementitious acids, as the effect of a too rapid conversion of the nitrogenized constituents of the blood and tissues into these highly oxygenized compounds.

The indications in the treatment of such cases are doubtless first, to prevent the too rapid formation of these compounds, by the use of a remedy—an organic acid for instance—which by its affinity for oxygen will most effectually prevent its excessive action upon the more important constituents of the blood and tissues; and secondly, to remove from the system these deleterious substances by the administration of an alkali which, by its union with the lithic and other acids, will produce soluble salts, such as can be more easily and readily taken up and discharged by the kidneys.

This two-fold object may be accomplished in some cases by the administration of salts, containing both the acid and the alkali, such as Tartrate of Potasa, or Lactate of Soda, and in others by the use of either the acid or alkali, according to the indications. Hence it is that the saline, alkaline and acid treatment for rheumatic affections, have each in turn had their advocates. It is a fact worthy of ote, that our views are sustained by two classes of writers, apparently opposed to each other—one contending for the alkaline, the other for the acid treatment of Rheumatism. Concerning the alkaline treatment, we find the following statement in a report of the treatment of disease in King's College Hospital, London:

"An unusual number of cases of Rheumatic fever have lately been admitted into Dr. Budd's ward; we noticed no less than six at the same time; with all of whom the alkaline treatment was successfully employed." This treatment, we may remark, consisted in the free use of bicarbonate of potash, both internally, and as a lotion upon the surface of parts affected.

Upon the use of organic acids, a recent number of the London Lancet contains the following statement, under the head of "Acute Articular Rheumatism treated with Lemon Juice."

"At Guy's Hospital, Dr. G. Owen Reece has introduced a method of treatment which amply deserves notice, both on account of its great simplicity, and the success which has attended it." In a subsequent number of the same periodical, Dr. Reece himself says in regard to this treatment: "My continued experience has but the more persuaded me, of the great value of Lemon Juice as a remedy for Rheumatism. Its action is sometimes most remarkable, causing cessation of pain and decrease of swelling and redness, such as we can rarely obtain with colchicum, even when administered in large and hazardous doses."

Should this simple medicine stand the test of time, as we believe it will, so as to enable the practitioner to avoid half poisoning his patients with colchicum, stupefying them with opium, or enervating them with calomel, a great boon will be conferred upon the consciencious physician, and upon suffering humanity.

Our views of this disease harmonize fully with those entertained by my colleague, Dr. Blaney, to whom is due the credit of having first suggested the use of Lactate of Soda, as a remedy for Rheumatism, with the view, as we suppose, of affecting directly or indirectly the removal of comparatively insoluble compounds, as for instance the phosphate of lime, by the production of two more soluble salts, such as the lactate of lime and phosphate of soda.

Another explanation of its mode of action which might be adopted is, that the lactate of soda constituted of an acid always present in the juice of flesh, combined with an alkali never absent from the blood, furnishes the two normal constituents which are deficient and therefore needed in this class of diseases; the lactic acid to combine with oxygen to form carbonic acid and water, and the soda to unite to form salts with the excess of acids, and thus destroy their deleterious properties and favor their excretion.

Whatever the true explanation of its mode of action, there can be no doubt of the value of this remedy in rheumatic affections, as might be shown, if time would permit, by the history of several cases treated by myself and others—one in particular under the care of Dr. Bird, which had remained unyielding under the most approved methods of treatment for years, was to all appearances permanently cured by the use of sour milk neutralized by the carbonate of soda.

Thus far we have endeavored to show that certain diseases are to be treated with the view to prevent oxydation, and that many of the remedies which have been found most efficient in effecting this object, are simple and common, composed frequently of substances, the absence of which in the blood and tissues, constitutes the pathology of the disease.

We will now present our views of the nature and treatment of another and opposite class, the pathology of which is, as we suppose, defective oxydation and an excess of carbonaceous material in the blood and tissues

The miasmattic diseases, such as Yellow, Billious, Remittent and Intermittent fevers, belong to this class, as we believe, for the following reasons. They originate always, in localities and under circumstances least favorable to oxydation, such as low, damp situations, covered with an atmosphere, saturated with moisture, and constituted in part of carbonic acid, sulphuretted and carburetted hydrogen, and other gases such as deteorate the air, both by their poisonous qualities and by their affinity for oxygen. The effect of such an atmosphere in preventing due oxydation, and hence the elimination of carbonaceous and other excrementitious matter, is made evident by the dark and sallow complexion of miasmatic patients, by post mortem appearances and by chemical analysis. So too the Symptoms, especially those indicating nervous derangement, are such as would naturally result from sluggish and irregular action of the brain and nerves, for want of a proper interchange of constituents by destructive oxydation and reparative deposition, without which no organ can properly perform its functions.

That this is true of the nervous system is proven by the well known fact, that the phosphates derived principally from nervous tissue are in excess in the urine during the progress of inflammatory affections of the brain or nerves; whilst on the other hand, there is a deficiency where defective mental and nervous action is indicated by a torpid intellect and blunted sensibility, facts clearly indicating that in one class there is excessive and in the other defective oxydation of nervous tissue.

As additional evidence of the correctness of these views, we will state that to our mind, many physiological as well as pathological phenomena, are more fully and satisfactorily explained by thus viewing cerebral and nervous action, as coincident with, if not dependent on oxydation.

The most remarkable characteristic of the nervous system, is a periodical tendency to action and repose, resulting evidently from circumstances which produce at one time a more, and at another a less rapid interchange of its constituents, causing the mind to vibrate with the regularity of a pendulum, between the activity of day, and the forgetfulness of night.

In order to explain the physiology of sleep, we must take into consideration the fact that during the day, a comparatively large amount of the inspired oxygen is required to sustain the action of the muscles, brain and nerves, and hence less is left free to combine with carbonaceous materials, which, therefore, go on accumulating till ultimately when night comes, their affinity for oxygen (being as in many other instances in proportion to quantity) is stronger than that of the albuminous compounds of which the nervous and muscular tissues are principally constituted; hence there is temporary suspension of mental and muscular action in the form of sleep; until, by the continued oxydation and elimination of this respiratory food it is reduced again to the point compatible with renewed mental and bodily action. The light and heat of day, which in favoring chemical action, have an opposite effect to that of the low temperature and darkness of night, influence doubtless these changes, but to what extent remains to be determined by future observation and experiment.

It would be an interesting and important point to determine, how far the above explanation of physiological phenomena would apply to certain pathological manifestations, such for instance as the periodical tendency to alternate excessive and defective action in Miasmatic Fevers, Intermittent Head-ache, Neuralgia and the like. If for instance it can be shown, that the cold stage of an ague, is but the effect of an accumulation of carbonaceous and other oxydable matters to such an exten as to suspend for a time the healthy action of the brain heart and blood-vessels, and that the subsequent febrile excitement is an effort of nature to throw off this superabundance of such matter, by temporary excessive chemical action, it follows that in this and all other diseases of the same class, the object of treatment should be to favor oxydation.

That this is in fact the true indication in the treatment of Miasmatic diseases, is shown by the unusual amount of highly oxydized effete matter discharged from the system during recovery, by the well known curative effects of pure air, and of remedial agents such as Iron, which favor oxydation by increasing the globular element of the blood.

It is more difficult to explain the modus operandi of certain other well known remedies, such as Quinine, Cinchonine, Strychnine, &c.; yet if we take into consideration their chemical nature in connection with certain chemco-physiological facts stated by Liebig, we can form an hypothesis, which, to say the least, will explain their mode of action better than any other yet offered.

"The blood-vessels and lymphatics," says this author, "contain an alkaline fluid, while the surrounding fluid, that of the flesh, is acid; the tissue of which the vessels are composed is permeable for the one or the other of these fluids. The constant occurrence of Chloride of Sodium and Phosphate of Soda in the blood," (having an alkaline reaction,) "and that of Phosphate of Potash and Chloride Potasium in the juice of flesh," (having an acid reaction,) "justify the assumption that both facts are altogether indispensable for the processes carried on in the blood and in the fluid of muscles."

"It is easy to foresee," to use the words of this distinguished chemest, "that a more exact study of the influence which alkalies, salts, and mineral acids exert on the respiratory process, in the normal state, must lead to the most beautiful and valuable results, in regard to their employment in various diseases." It is a remarkable fact worthy to be taken into consideration in connection with this subject, that as a general rule the organic acids are the most efficient remedial agents in rheumatic affections, and other diseases of that class, in which, as we contend, there is excessive oxydation; whilst those of the opposite character, as the Miasmatic Fevers, are treated most effectually with the vegetable alkalies, such as Quinine, Cinchonine, &c., either alone or in combination, not with the organic, but with the inorganic acids.

Whether these remedial agents favor oxydation by combining with, and removing acids, or by what chemists call catalysis, is a question of much physiological interest, but of little practical value, as affecting the general principle which the above facts seem to establish, that alkalies, especially those derived from the vegetable kingdom, are as a class, the proper remedies for the diseases under consideration.

Chloride of Sodium is a well known simple article, in common use, which, with our old notions of heroic treatment, would never have been suspected of possessing medicinal properties, yet modern efforts to devise means by which to avoid poisoning our patients, have resulted in the discovery that common salt may be and is used, for other and more important purposes than that of making food palatable, as will appear from the following statement taken from a recent periodical, concerning the use of this new and simple remedy.

"Prof. Piorry, in reporting to the Academy of Medicine (Paris) upon the proposed use of table salt in intermittent fevers, states that if administered in doses of two table-spoonfulls it will not only arrest the disease, but also exert upon the spleen as marked effect as quinine does. In twelve cases of intermittent fever, the salt uniformly arrested the paroxysms, and lessoned very materially the size of the spleen. The spleen was also found to diminish when the remedy was given, in cases of typhoid fever."

Before meeting with the above, our attention had been called to this remedy, by my colleague Dr. Brainard, who in view of its well known effects in preventing decay and the destruction of blood globules, was induced to administer salt, in some cases of Pneumonia, attended with great prostration and copious brick-dust expectoration. The effect of this treatment in relieving all the unpleasant symptoms, and especially in changing the character of the sputa, was prompt and satisfactory.

More recently other cases of the same, and some of a different character, have been treated with this remedy in the Illinois General Hospital under our direction, with results equally satisfactory.

A case of Jaundice, for instance, in its worst form, improved rapidly under the use of mutton broth, nearly saturated with salt. Another most unpromising case of an emigrant direct from Germany with ship fever symptoms, improved more rapidly and got well sooner, under this treatment, than any case of the kind I have ever seen of the same severity.

In order to show the importance of this substance as an article of diet, and hence its value as a remedial agent, we will give one other short quotation from Liebig's valuable work on the Chemistry of Food

"In inland countries the food does not contain common salt enough to produce the phosphate of soda necessary for the formation of the blood, then more salt must be added to the food. From the common salt is produced in this case, by mutual decomposition with phosphate of potash, or with earthy phosphates, the phosphate of soda of the blood. That the phosphate of soda is indispensable to the normal constitution of the blood, and that the processes which go on in that fluid cannot be replaced by phosphate of potash, seems to me, to be an opinion justified by the properties of these two salts."

Such being its physiological importance, the conclusion, as it seems to me, is inevitable, that it must be an efficient remedy in certain pathological conditions; such, for instance, as an undue accumulation of acid, for want of oxygen to burn up the lactic, or of soda to neutralize the phosphoric and other acids.

The acids in the blood and tissues, according to the author above quoted, are shared by the alkalies soda, potash, &c., so that the amount of free acid present must stand in a definite relation to the quantity of the base; hence where there is an excess of free lactic acid from defective oxydation, or of others from undue accumulation, common salt may be administered with the view of providing soda to neutralize some and combine with others, to form easily eliminated soluble salts.

It would appear, then, that the diseases in which this remedy is indicated, are those in which there is an excess of acids, and in which a sallow and dark complexion and brick-dust expectoration indicate the escape of the coloring matter of the blood, from ruptured globules; which, as has been shown by numerous experiments, are destroyed by acids, and preserved by saline and alkaline solutions.

We have been thus particular in expressing our views of the action of the Chloride of Sodium, with the hope that others may be led to try its effects, believing, as we do, that this the most essential perhaps of the inorganic constituents of food, and we may say of blood, will prove ultimately one of the most valuable and efficient of the remedial agents.

In the preceding remarks, we have attempted to combine and harmonize a few of the most important modern discoveries and recently observed facts, with the view principally of showing the importance of having the science and practice of medicine keep pace together, and go hand in hand in the march of improvement, in order that each may by turns support and guide the other through the obseure and intricate path leading in our profession, to knowledge and truth.

Since the discovery of the important relations existing between vegetation and the chemical constitution of soil, no well informed agriculturist would think of bettering the condition or of promotiug the growth of a sickly tree, in any other way than by removing unnatural and exuberent bark and branches, and supplying its roots with an earth containing all the proper constituents for its body and fruit; neither should we with our present knowledge of the dependence of healthy action upon proper nutrition, neglect to apply in practice each newly acquired fact, by which we can the better determine how to harmonize vital action, by adding healthy and abstracting morbid constituents of the body in a manner as near as possible as nature dictates in her unaided and healthy performance of those functions.

Having now occupied all the time we feel at liberty to take on the present occasion, with what is in truth nothing more than a brief and imperfect numeration of a few only of the facts and arguments which might be adduced in support of our views upon several subjects, each of which might properly have claimed the whole of our attention, and having as we trust, to some extent at least, sustained our position, we will now, in conclusion, renew the assertion, that in our opinion the time is not far distant when the truly scientific physician will use as remedies such substances only as help to constitute in health, the solids and fluids of the body.

Even now, the best informed and thinking men of our profession, are beginning to practice in accordance with these views, learning as they are from reason and daily experience, that the best materials with which to repair a broken fabric, are those used in its construction by the Great Master Builder.

